# A review study of fetal circulatory models to develop a digital twin of a fetus in a perinatal life support system

**DOI:** 10.3389/fped.2022.915846

**Published:** 2022-09-21

**Authors:** Bettine G. van Willigen, M. Beatrijs van der Hout-van der Jagt, Wouter Huberts, Frans N. van de Vosse

**Affiliations:** ^1^Cardiovascular Biomechanics, Biomedical Engineering, Eindhoven University of Technology, Eindhoven, Netherlands; ^2^Obstetrics and Gynaecology, Máxima Medical Centre, Veldhoven, Netherlands; ^3^Signal Processing Systems, Electrical Engineering, Eindhoven University of Technology, Eindhoven, Netherlands; ^4^Department of Biomedical Engineering, CARIM School for Cardiovascular Diseases, Maastricht University, Maastricht, Netherlands

**Keywords:** fetal cardiovascular system, mathematical models, review, digital twin, perinatal life support system

## Abstract

**Background:**

Preterm birth is the main cause of neonatal deaths with increasing mortality and morbidity rates with decreasing GA at time of birth. Currently, premature infants are treated in neonatal intensive care units to support further development. However, the organs of, especially, extremely premature infants (born before 28 weeks of GA) are not mature enough to function optimally outside the womb. This is seen as the main cause of the high morbidity and mortality rates in this group. A liquid-filled incubator, a so-called PLS system, could potentially improve these numbers for extremely premature infants, since this system is designed to mimic the environment of the natural womb. To support the development and implementation of such a complex system and to interpret vital signals of the fetus during a PLS system operation, a digital twin is proposed. This mathematical model is connected with a manikin representing the digital and physical twin of the real-life PLS system. Before developing a digital twin of a fetus in a PLS system, its functional and technical requirements are defined and existing mathematical models are evaluated.

**Method and results:**

This review summarizes existing 0D and 1D fetal circulatory models that potentially could be (partly) adopted for integration in a digital twin of a fetus in a PLS system based on predefined requirements. The 0D models typically describe hemodynamics and/or oxygen transport during specific events, such as the transition from fetus to neonate. Furthermore, these models can be used to find hemodynamic differences between healthy and pathological physiological states. Rather than giving a global description of an entire cardiovascular system, some studies focus on specific organs or vessels. In order to analyze pressure and flow wave profiles in the cardiovascular system, transmission line or 1D models are used. As for now, these models do not include oxygen transport.

**Conclusion:**

This study shows that none of the models identified in literature meet all the requirements relevant for a digital twin of a fetus in a PLS system. Nevertheless, it does show the potential to develop this digital twin by integrating (parts) of models into a single model.

## Introduction

### Clinical background

Preterm birth is the main cause of neonatal deaths (about 35% of all cases) and accounts for 16% of child mortality under 5 years [UNICEF report; (1)]. The morbidity (2, 3) and mortality (4) rate increases with decreasing GA at the time of birth. To stratify the severity of preterm birth, three subgroups are defined: extremely preterm (< 28 weeks), very preterm (28–32 weeks), and late preterm (32–37 weeks) (WHO report; (5)). Globally, incidence rate of preterm birth for these subgroups is 4.1, 11.3, and 84.7%, respectively, (6) with an all-cause mortality incidence rate ratio of 1.91, 1.64, and 1.31, respectively, relative to the full-term birth (4). For extremely preterm born infants of 24–26 weeks GA, the chance of survival to 2 years is about 65–80% depending on GA (7).

Currently, medical care for premature infants is focused on supporting and monitoring temperature, respiration, cardiac function, oxygen saturation, and brain activity. However, supporting neonatal physiology may not be sufficient to treat premature infants. The transition from fetus to neonate requires a change in oxygen and nutrients source (lungs and gastrointestinal tract instead of placenta) and adaptation of the cardiovascular system (closing of shunts) (see Section Fetal cardiovascular physiology) (8). Therefore, this transition may result in life-long complications for premature infants when maturity of the organs has not been reached yet (9). These complications include respiratory, gastrointestinal, neurological, feeding, visual, and hearing problems (6, 10–13). Between 40 and 60% of the extremely preterm survivors experience major morbidity in the first year with about 75% when born at 23 weeks to about 45% when born at 26 weeks of GA (3). The review of (14) shows increased neuropsychiatric disorders for extremely preterm and very preterm compared with full-term born infants. Furthermore, an increase in moderate and severe disabilities at 18 years is seen by decreasing GA at time of birth from 5.6 and 3.7%, respectively, for full-term born infants, to 10.6 and 11.8%, respectively, for infants born at 24 weeks GA (15).

The short- and long-term health problems of these premature infants have enormous economical consequences. The initial hospital costs of live births are inversely associated with GA ranging from approximately $111,000 to $577,000 for 24 weeks to $900 to $7,000 for full-term infants (16). In addition, the estimated incremental costs of very and extremely preterm surviving to 18 years is substantial higher (~$96,000 and ~$147,000, respectively) compared with a full-term infant (~$35,000) (15).

The current healthcare system seems to have reached its limits regarding the support of neonatal physiology-based intensive care. Therefore, current research investigates a shift in medical care for extremely premature infants from supporting neonatal to remain fetal life *ex utero* in order to increase the chance of survival, reduce morbidity, and reduce the social economical costs (17–19). To achieve this, the transition from fetus to neonate needs to be postponed until organs have reached a sufficient level of maturity. This could be achieved with the development of a novel liquid-filled incubator for extremely premature infants, a PLS system (19–21). Such an incubator is designed to mimic the environment of the natural womb and is being developed to allow the fetal lungs to remain filled with liquid, while the umbilical cord is connected to an artificial placenta.

The PLS system will consist of an artificial placenta to supply nutrients and oxygen, a liquid-based environment with artificial amniotic fluid including an amniotic dialyzer, sensors to measure fetal vital signals, and a monitoring and model-based decision support system to interpret the signals and suggest interventions. All these components have to be tailored to the requirements of the PLS system (17). For instance, to avoid damaging of blood, no blood pumps should be used in the fetal-artificial placenta circulation (19), which implies that a low resistance, low volume artificial placenta is required. Furthermore, monitoring of the fetus should be possible despite the design choices for the system, e.g., regarding signal permeability through the PLS system walls.

Therefore, to develop and implement such a liquid-based incubator, different design choices have to be considered and knowledge of multidisciplinary fields have to be integrated into one system. A virtual representation of the PLS system could support this integration by testing and verifying scientific hypotheses, and similarly by predicting the interaction of mechanical components and their effect on the fetus. Ultimately, such a model can support clinical decision making by interpreting measured vital signals and estimating immeasurable but important parameters when the PLS system is introduced in the clinic. Furthermore, mathematical models could provide understanding of the underlying mechanisms to determine the physiological state of the fetus. Currently, the knowledge about the physiological state of the fetus is mainly based on non-invasive measurements, when anatomical and genetic disorders are ruled out. Ultrasound is used to measure growth, heart rate, and the amniotic fluid volume. When fetal growth restriction is suspected, Doppler ultrasound is used to measure blood velocities in the umbilical cord (22, 23) and/or one of the cerebral arteries (24) to assess Doppler indices. When the Doppler indices are abnormal, cardiotocography is performed to assess fetal heart rate and uterine activity. Bases on these findings, labor could be induced when the cardiotocogram is abnormal. Maternal blood pressure is measured to rule out hypertensive disease of pregnancy. These measurements are indirect data to determine whether placental capacity is sufficient in order to provide the fetus with enough nutrients and oxygen for growth and development. As all these measurements are clinically used to determine the physiological state of a fetus, it is of importance to understand the underlying mechanisms. For this, the use of mathematical models will be indispensable, not only as part of the development and implementation strategy of PLS system, but also as decision support tool during medical treatment. In the development phase, the digital twin will particularly be used to leverage our knowledge of the fetal physiology and to guide the development and implementation of a PLS system. To this end, the digital twin can be connected to a physical twin, i.e., a fetal manikin, to simulate fetal responses during training sessions. Once the PLS system has been implemented for clinical treatment, the digital twin model can also be used as decision support tool in clinical practice to predict the effect of interventions. For the development of the digital twin of a fetus in a PLS system, existing models are evaluated against the digital twin functional and technical requirements, which are specified in the next sections.

### Functional requirements

The purposes of the digital twin required to support the development and implementation of a PLS system are:

1. **Gaining knowledge:** To develop a PLS system, extensive knowledge about the fetal physiology is necessary. However, this knowledge is (yet) insufficient mainly due to scarcely available data, as to protect mother and infant, data acquisition is limited by non- or minimal invasive tests during pregnancy. Hence, current data often consist of indirect measurements and/or animal data. Furthermore, the few invasive human fetal data available, have been derived from fetuses that required an intrauterine intervention. As a result, often assumptions and hypotheses are used to estimate the wellbeing of the fetus non-invasively. By testing these hypotheses, the digital twin should help gaining the necessary knowledge of the physiology of the fetus to create an optimal environment for fetal development.
2. **Optimizing components:** To analyze the interaction of the mechanical components of a PLS system with the fetus, the digital twin must be able to simulate the mechanical and fluid dynamic functioning of these components. Hence, the digital twin should be able to optimize the desired effect of the components on the fetal physiology.
3. **Monitoring:** When a PLS system is implemented in the clinic, the digital twin should be able to monitor and to interpret vital signals during a PLS system operation. These vital signals include heart rate, blood flow over the cardiac valves, cerebral and peripheral oxygen saturation, and blood pressure and flow in the important vessels (aorta, umbilical cord, and vena cava) and fetal cardiovascular shunts (ductus venosus, ductus arteriosus, and foramen ovale).
4. **Decision support:** The digital twin should serve as patient-specific decision support tool for clinicians. The tool should advice the user about the settings of a PLS system or even support interventions by fetal distress.
5. **Training of clinicians:** Before implementation of a PLS system, clinicians have to be trained to transfer a fetus from the natural womb to the system. For training purposes, the digital twin connected with a manikin can provide real-life simulation scenarios.
6. **Digital twinning:** For a mathematical model to be defined as digital twin, the mathematical model should be dynamically paired with the real-life fetus in the PLS system. In other words, the mathematical model should integrate the measurements of the fetal vital signals and provide decision support. The measurements of the vital signals include oxygen saturation in cerebral microcirculation, estimation of fetal weight, fetal electrocardiogram, and blood flow and pressure through the umbilical cord.

### Technical requirements

To satisfy the above-mentioned functional requirements, the digital twin should meet technical requirements described in this paragraph. The cardiovascular system is the transport system for oxygen, nutrients, and metabolic waste products. Hence, it is a key facilitator of growth and development. In addition, the PLS system sustains fetal physiology. Therefore, this paper and also the set of requirements in this section are mainly focused on the fetal cardiovascular system including oxygen transport and fetal growth. Hence, the nutrient utilization and hormone pathways are excluded from this study.

1. **Cardiovascular system:** The digital twin should describe the pressure and flow relations in the important vessels in the cardiovascular system to analyze the hemodynamic state of the fetus. These vessels include cerebral and umbilical vessels, aorta, vena cava, and shunts (ductus arteriosus, ductus venosus, and foramen ovale).
2. **Closed-loop:** A closed-loop of the prenatal circulation is required to simulate the entire fetal cardiovascular system including the shunts and venous return.
3. **Pulsatile driving force:** The digital twin will be verified on available clinical data. These data consist mainly of blood velocities measured by Doppler ultrasound, fetal heart rate, and growth. To simulate blood velocity waves, the cardiovascular system should be provided with a pulsatile pressure or flow source in physiological or pathological range. This could be generated by a fetal cardiac contraction model or a prescribed profile.
4. **Wave propagation and reflections:** In order to propagate these pulsatile pressure and flow waves throughout the entire cardiovascular system, wave propagation and reflections of pressure should be incorporated in the digital twin. Furthermore, the shape of the pressure and flow waves have potential diagnostic value.
5. **Maternal-fetal gas exchange:** Maternal-fetal gas exchange takes place in the placenta. Placental insufficiency could lead to lower oxygen saturation in the fetus. Therefore, it is of importance that the digital twin contains maternal-fetal gas exchange mimicking oxygen transport during physiological and pathological conditions.
6. **Baro- and chemoreceptor reflex:** Baro- and chemoreceptor reflex are part of the regulatory system to maintain homeostasis. Furthermore, redistribution of the cardiac output when the fetus is in distress is regulated by these reflexes. Therefore, the digital twin should contain baro- and chemoreceptor reflexes. Noteworthy, the regulatory system of extremely premature infants is different from adults and full-term fetuses (25).
7. **Fetal growth:** The fetus growths from about 700 g at 24 weeks to 1,200 g at 28 weeks GA (26). Hence, the digital twin has to be able to predict fetal growth and its impact on the circulation during physiological and pathological conditions.
8. **Real-time:** Computational time should be pseudo-real-time such that the digital twin can be used as clinical decision support tool when a PLS system is introduced in the clinic. The digital twin should be able to run in a local clinical setting as patient data cannot leave the hospital and, hence, the digital twin should be integrated in an advanced personal computer or dedicated workstation that can be located on the ward. Therefore, 3D modeling is most likely not feasible. However, when a particular part of the digital twin requires 3D modeling to properly describe its function, meta-models should be developed allowing the desired order of detail with less computational expense.

**Figure 1:**
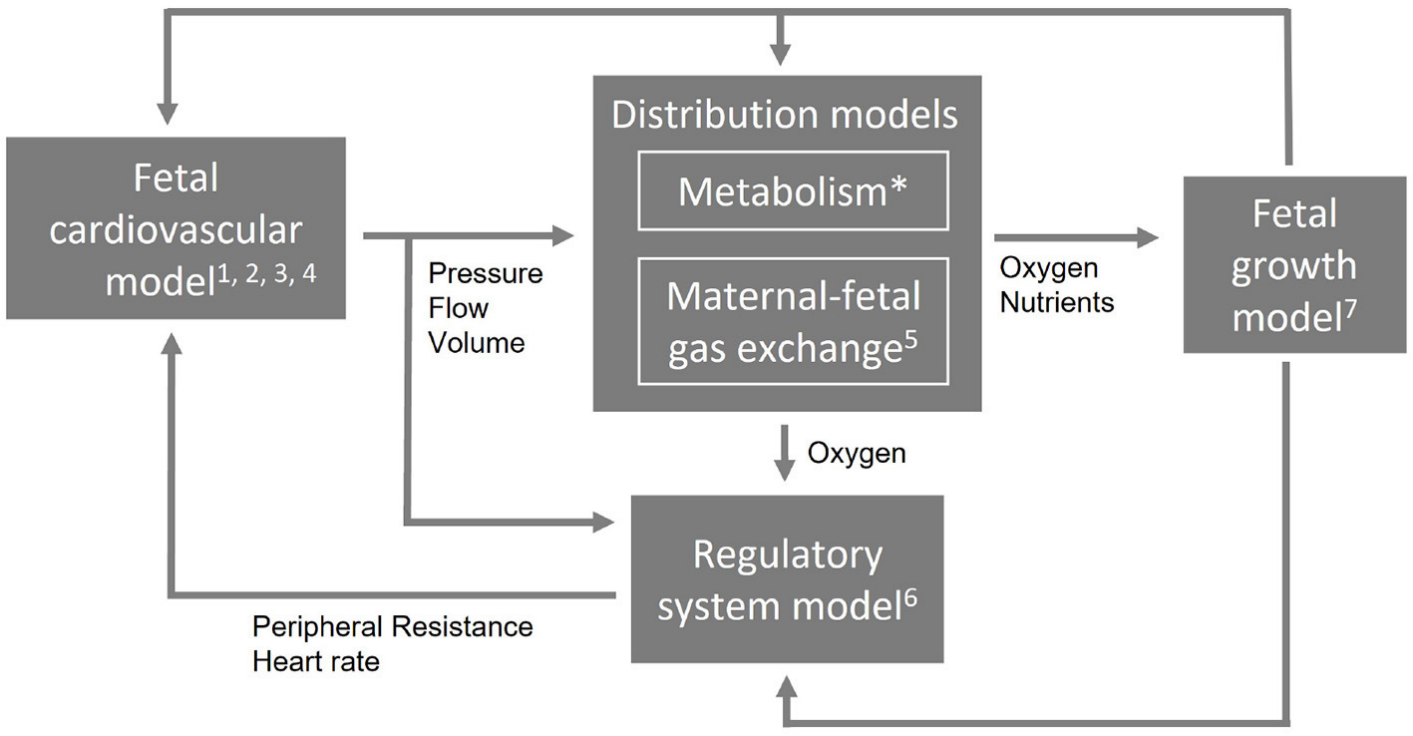
An example of a modular arrangement. The numbers refer to the numbering of the technical requirements (Section Technical requirements). The first four requirements are applicable to the cardiovascular model. Metabolism is not included in this study (*) and the computational time applies to the entire model (not shown).

These requirements demand for a computational model that is multiscale and multilevel in order to simulate different length and time scales and complexity. To allow such a complex combination of multiscale and multilevel modeling approach and to remain flexibility to extend, reduce, or adapt parts of the model, it needs a modular arrangement. Figure 1 shows an example of a modular arrangement. In this example, the fetal cardiovascular model is a closed-loop model (2. closed-loop) containing the description of the vessels (1. cardiovascular system) driven by a pulsatile force (3. pulsatile driving force) creating realistic pressure and flow waves (4. wave propagation and reflections). The resulting pressure, flow, and volume in the vessels are the input of the metabolism model (not included in this study) and maternal-fetal gas exchange model (5. maternal-fetal gas exchange). Based on the oxygen saturation resulting from the maternal-fetal gas exchange model, feedback is provided by the regulatory system model (6. baro- and chemoreceptor reflex) to the fetal cardiovascular model by changing the peripheral resistance and heart rate accordingly to the fetal needs. Furthermore, oxygen and nutrients are necessary for fetal growth (7. fetal growth) leading to adaptation of parameter settings in the cardiovascular, distribution, and regulatory system models. Section Model integration elaborates on the integration of model functionalities on multiscale and multilevel.

### Existing fetal circulatory mathematical models

Part of the current research is based on the complete circulation implemented as a LPM to describe global distributions of flow, volume, and pressure [e.g., (27–31)]. Others are focusing in more detail, by means of 1D or 3D modeling, on a part of the circulation. For instance, to evaluate forward and reflected waves or pulsatile flow in the umbilical artery [e.g., (32–38)], arterial side [e.g., (39–42)], aortic coarctation [e.g., (43)], umbilical vein [e.g., (44)], or the feto-placental arterial network [e.g., (45, 46)]. In order to simulate a complex system like the fetal cardiovascular circulation with oxygen transport and fetal growth, we are not solely looking for global distributions of blood volume, pressure, and flow or parts of the fetal physiology, but for a single model that describes all requirements (Sections Functional requirements, Technical requirements). Within a single model, the fetal cardiovascular circulation can be assessed as an entity and not as isolated parts. Therefore, the digital twin should integrate all necessary functionalities of existing mathematical models in a coherent description of the fetal cardiovascular system including fetal growth and oxygen transport.

### Aim

The aim of this paper is to provide a review evaluating how existing models meet (part of) the digital twin model requirements in order to propose a strategy for the development of this digital twin of a fetus in a PLS system.

### Outlook

Building upon the predefined requirements (Sections Functional requirements, Technical requirements), the fetal cardiovascular physiology (Section Fetal cardiovascular physiology) and the physics to describe this physiology (Section 0D and 1D circulatory models) are presented. Subsequently, the search query is defined as well as the assessment method for the identified models (Section Assessment of existing models). The literature survey leads to an overview of 0D models (Section 0D models) and 1D models (Section 1D models). Next, these mathematical models are discussed (Section Discussion), while considering the technical requirements (Section Technical requirements) and how the models can be integrated. In addition, the model proposal for a digital twin of a fetus in a PLS system (Section Model proposal: A digital twin of a fetus in a PLS system) is presented and the limitations of this study are given. Finally, a conclusion summarizes the insights obtained and their implication for the development of a digital twin of a fetus in a PLS system (Section Conclusion).

## Fetal circulatory models

### Fetal cardiovascular physiology

**Figure 2:**
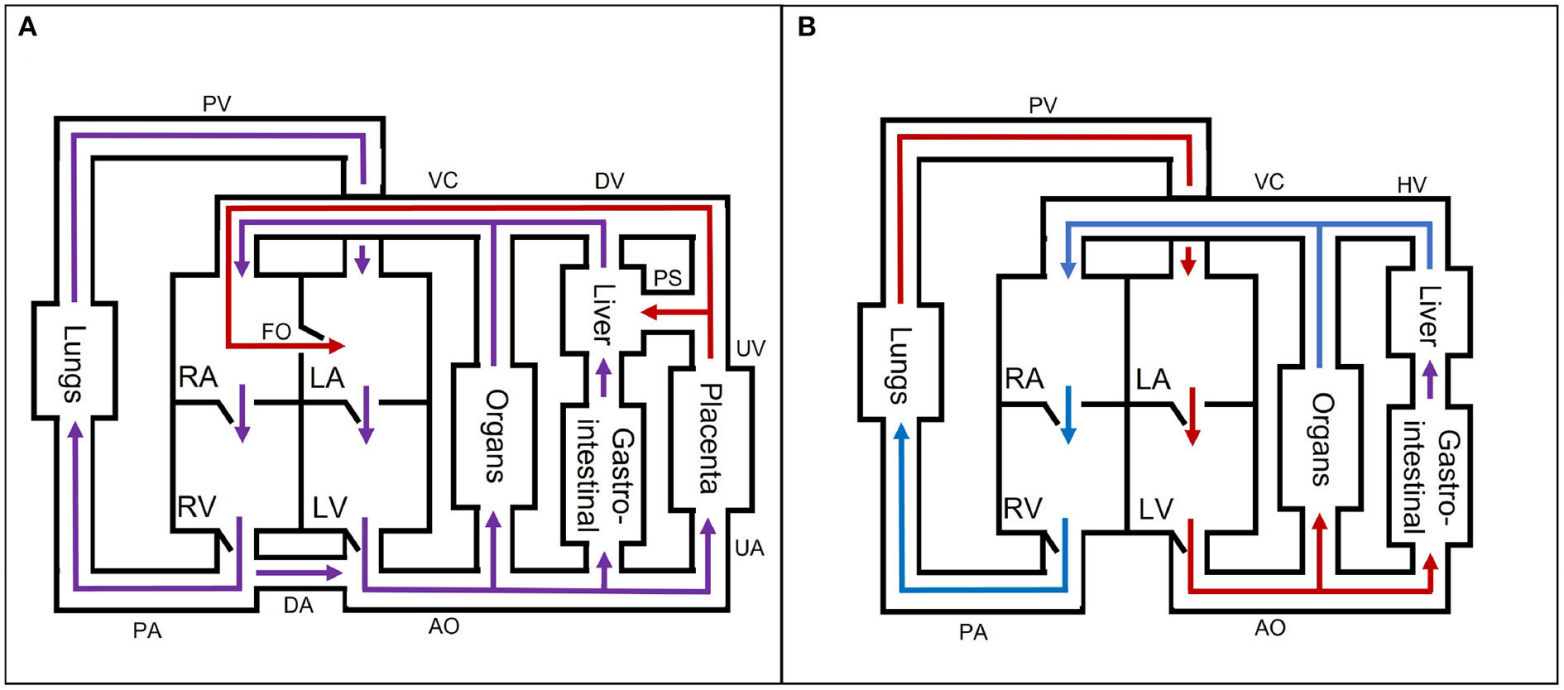
A simple schematic overview of the **(A)** fetal and **(B)** neonatal circulation with oxygen- and nutrient-rich (red), oxygen- and nutrient-poor (blue), and oxygen- and nutrient-rich and -poor mixed (purple) blood flow direction. AO, aorta; DA, ductus arteriosus; DV, ductus venosus; FO, foramen ovale; HV, hepatic veins; LA, left atrium; LV, left ventricle; PA, pulmonary arteries; PS, portal sinus; PV, pulmonary veins; RA, right atrium; RV, right ventricle; UA, umbilical arteries; UV, umbilical vein; VC, inferior and superior vena cava. The block “organs” represents all other organs, which are not separately mentioned in the figure.

The fetal cardiovascular system (Figure 2A) deviates from the adult cardiovascular system (Figure 2B) as the fetus receives oxygen and nutrients from the placenta instead of the lungs and gastrointestinal tract, respectively. The oxygen- and nutrient-rich blood from the placenta flows *via* the umbilical vein to the portal sinus and the ductus venosus. The portal sinus supplies the liver with oxygen- and nutrient-rich blood *via* the portal vein. The oxygen- and nutrient-rich blood in the ductus venosus enters the inferior vena cava and flows into the right atrium. In the right atrium, oxygen- and nutrient-rich blood flows preferentially to the left atrium *via* the foramen ovale. A smaller part of the right arterial blood flows to the right ventricle. In the right ventricle, a small part flows to the pulmonary arteries to supply the lungs. The rest of the blood mixes with the aortic blood *via* the ductus arteriosus. Blood from the foramen ovale and pulmonary veins in the left atrium flows into the left ventricle to the rest of the organs and enters ultimately in the two umbilical arteries that brings the blood back to the placenta for nutrient uptake and re-oxygenation. After birth, the cardiovascular system undergoes multiple changes to switch from placental to pulmonary oxygenation. This cascade is initiated by, among other things, the first breaths after birth. As a result, the shunts (ductus venosus, foramen ovale, and ductus arteriosus) will close in the following hours. In addition, the umbilical cord is cut, thus causing closure of the umbilical vessels. These changes all contribute to the transition from fetal to neonatal cardiovascular physiology. For an extensive description of the fetal cardiovascular system, the reader is referred to (8).

### 0D and 1D circulatory models

#### Cardiovascular system

The motion of blood through the fetal cardiovascular system is governed by the Navier-Stokes equations. These partial differential equations are based on the balance of mass (1) and momentum (2). When assuming an incompressible fluid, the Navier-Stokes equations are given by:


(1) $$\begin{array}{l} \frac{\partial \rho }{\partial t}+\nabla \cdot \left(\rho \text{u}\right)=0, \end{array}$$

(2) $$\begin{array}{l} \frac{\partial }{\partial t}\left(\rho \text{u}\right)+\nabla \cdot \left(\rho \text{u}⊗\text{u}\right)=-\nabla p+\nabla \cdot \text{τ}+\rho \text{f}, \end{array}$$

with ρ the blood density $\left[\frac{g}{m^{3}}\right]$, **u** the blood velocity $\left[\frac{m}{s}\right]$, *p* the hydrostatic pressure [*Pa*], τ the shear stress tensor [*Pa*], and *f* body accelerations $\left[\frac{m}{s^{2}}\right]$, such as gravitational forces. When (1) and (2) are integrated over the cross section *A*, the following formulas are obtained:


(3) $$\begin{array}{l} \underset{A\left(t\right)}{\iint }\left[\frac{\partial \rho }{\partial t}+\nabla \cdot \left(\rho \text{u}\right)\right]dA=0, \end{array}$$

(4) $$\begin{array}{l} \underset{A\left(t\right)}{\iint }\left[\frac{\partial }{\partial t}\left(\rho \text{u}\right)+\nabla \cdot \left(\rho \text{u}⊗\text{u}\right)\right]dA=\underset{A\left(t\right)}{\iint }\left[-\nabla p+\nabla \cdot \text{τ}+\rho \text{f}\right]dA. \end{array}$$

1D wave propagation models can subsequently be obtained after applying Reynolds transport theorem (47), assuming cylindrical symmetry, and applying no-slip condition on the wall. This results in a blood velocity predominantly along the longitudinal or central axis (z-direction) that varies perpendicular to this axis (r-direction). In other words, velocity is zero with respect to the wall velocity. As the velocity is predominantly in z-direction, the velocity in r-direction and circumferential direction (θ-direction) is neglectable. Hence, the pressure and flow in the vessels are dependent on the z-direction and time (*p*(*z, t*) and *q*(*z, t*), respectively). Subsequently, (3) results in (5) by dividing by ρ and multiplying by *A*:


(5) $$\begin{array}{l} \frac{\partial A}{\partial t}+\frac{\partial q}{\partial z}=0, \end{array}$$

with *q* the blood flow. Using Reynolds transport theorem, (4) results in:


(6) $$\frac{\partial q}{\partial t}+\frac{\partial }{\partial z}\left(\delta \frac{q^{2}}{A}\right)=\frac{A}{\rho }\left(-\frac{\partial p}{\partial z}+\frac{2}{r}\tau_{z}\right)+Af_{z},$$

with τ_*z*_ the wall shear stress [*Pa*], *f*_*z*_ body accelerations $\left[\frac{m}{s^{2}}\right]$, δ a variable to define the velocity profile [−] (48, 49), r the radius. Subsequently, to solve (5) and (6), a constitutive relation has to be defined to give a relation between *p* and *A*. For elaborate description of the derivations, the reader is referred to the review of (48). When (5) and (6) are integrated over the length *z*, formula to describe 0D models are derived:


(7) $$\begin{array}{l} \underset{z\left(t\right)}{\int }\left[\frac{\partial A}{\partial t}+\frac{\partial q}{\partial z}\right]dz=0, \end{array}$$

(8) $$\int_{z(t)}\left[\frac{\partial q}{\partial t}+\frac{\partial }{\partial z}\left(\delta \frac{q^{2}}{A}\right)\right]dz=\int_{z(t)}\left[\frac{A}{\rho }\left(-\frac{\partial p}{\partial z}+\frac{2}{r}\tau_{z}\right)+Af_{z}\right]dz.$$

Substituting the area compliance $C_{A}=\frac{\partial A}{\partial p}$ in (7) results in:


(9) $$\begin{array}{l} C\frac{\partial p}{\partial t}+\text{Δ}q=0, \end{array}$$

with C the vessel compliance $\left[\frac{m^{3}}{Pa}\right]$ (*C* = *C*_*A*_Δ*z*). When the non-linear convection term $\left(\frac{\partial }{\partial z}\left(\delta \frac{q^{2}}{A}\right)\right)$ and the external forces (**f_*z*_**) are neglected and the viscous term (τ_*z*_) is approximated with Poiseuille shear stress, a 0D formulation of the 1D momentum balance (6) is derived:


(10) $$\begin{array}{l} L\frac{\partial q}{\partial t}=-\text{Δ}p+Rq, \end{array}$$

with L the inertia $\left[\frac{Pa\cdot s^{2}}{m^{3}}\right]$ and R the resistance $\left[\frac{Pa\cdot s}{m^{3}}\right]$.

#### Pulsatile driving force

The models with a pulsatile input contain a fetal cardiac module (time varying elastance model or one fiber model) or a prescribed flow or pressure profile. The time varying elastance model describes the pressure in a cardiac cavity (*p*_*cav*_) with a variable compliance, which is based on the minimum (*E*_*min*_) and maximum elastance (*E*_*max*_) of the heart muscle in $\left[\frac{Pa}{m^{3}}\right]$ and the instantaneous volume of the cavity *V*_*cav*_ (50). The cavity pressure is given by:


(11) $$\begin{array}{l} p_{cav}\left(t\right)=\left(\left(E_{max}-E_{min}\right)a\left(t\right)+E_{min}\right)\left(V_{cav}\left(t\right)-V_{cav,0}\right), \end{array}$$

with *V*_*cav*, 0_ the cavity volume at zero pressure [*m*^3^] and *a*(*t*) the time dependent activation function of the contraction with a value between 0 and 1 [−]. The underlying phenomena of cardiac contraction are better described by the one fiber model, as this model relates *p*_*cav*_ to the cavity volume *V*_*cav*_, myocardial wall volume (*V*_*wall*_), and myocardial fiber stress (σ_*f*_), with the latter depending on the fiber length, activation time, and sarcomere shortening velocity (51, 52):


(12) $$\begin{array}{l} p_{cav}\left(t\right)=\frac{1}{3}\sigma_{f}\ln\left(1+\left(\frac{V_{wall}}{V_{cav}}\right)\right). \end{array}$$

This model is based under the assumption that the stress in the myocardial fibers is homogeneous, which allows modeling of a single-fiber.

#### Maternal-fetal gas exchange

The transport of oxygen expressed as the rate in change of oxygen in an organ $\left(\frac{d\left(c_{O_{2}}V\right)}{dt}\right)$ is the sum of the convective transport to and from the organ (*Q*_*C*_), the metabolic consumption of the organ (*Q*_*M*_), and diffusive exchange (*Q*_*D*_) (53):


(13) $$\begin{array}{l} \frac{d\left(c_{O_{2}}V\right)}{dt}=Q_{C}+Q_{D}-Q_{M}, \end{array}$$

with *V* the volume of the organ [*mL*], the oxygen fluxes (*Q*_*c*_, *Q*_*M*_, and *Q*_*D*_) in $\left[\frac{mL^{o_{2}}}{s}\right]$, and *c*_*O*_2__ the oxygen concentration $\left[\frac{mL^{o_{2}}}{mL^{blood}}\right]$.

### Assessment of existing models

Section Technical requirements mentioned the technical requirements of the digital twin of a fetus in a PLS system. To find eligible models that (partly) satisfy the technical requirements, a literature search^1^ is executed on fetal cardiovascular models including fetal growth and oxygen transport. Subsequently, each paper is assessed based on the requirements as summarized in Table 1. For the fetal growth requirement, the age and/or weight is included as well. Furthermore, pulsatility can be obtained by a cardiac model mimicking the contraction of the heart, such as the TVEM (50) and OFM (51) or by a prescribed flow or pressure profile (PP). The computational time is not included as many papers did not mention this. This study is limited to models in 0D and 1D, and the identified models are organized accordingly. For an elaborate description about the contribution of 0D and 1D modeling to describe cardiovascular systems, the reader is referred to (54).

**Table 1:** Technical requirements and their assessment.

**Technical requirements**	**Assessment**
Cardiovascular system	Represented vessels
Closed loop	Yes or no
Pulsatile	Yes; TVEM, OFM, PP or no
Wave propagation	Yes or no
Maternal-fetal gas exchange	Yes or no
Fetal growth	Yes or no; age and/or weight
Baro- and chemoreceptor reflex	Yes or no

OFM, one fiber model; PP, prescribed pressure or flow profile; and TVEM, time varying elastance model.

### 0D models

Describing the fetal cardiovascular system as a 0D model is intensively explored in literature. These models mostly describe the cardiovascular system as analog of an electrical circuit. In this study, two different 0D model types are described; lumped parameter models and transmission line models. A lumped parameter model consists of several compartments describing the microcirculation of an organ, a vessel, or part of a vessel. The number of compartments depends on the research question. As homogeneity is assumed within a compartment, global distributions of flow, pressure, and volume can be examined. Typically, the local effects of wave propagation and reflections in larger vessels can not be analyzed in the coarse representation of the cardiovascular system. Nevertheless, uniform distribution of the variables still provide a general overview of the functionalities of the fetal cardiovascular system. Furthermore, lumped parameter models are used to provide boundary conditions for higher dimensional models. Transmission line models are distributed lumped parameter models, meaning that the compartments are described with multiple segments assuming non-homogeneity within a compartment. Therefore, transmission line models, in contrast to lumped parameter models, allow simulation of forward and backward pressure and flow waves. To capture those waves, as a rule of thumb, the length of these segments should be 10 times smaller than the smallest wavelength, as a pressure signal has approximately a maximal frequency of 10 Hz (55). The next sections provide an overview of the currently existing lumped parameter and transmission line models. Supplementary Table 1 gives an overview of the assessment based on the technical requirements.

#### Lumped parameter models

Huikeshoven et al. (27) developed the first closed-loop fetal cardiovascular lumped parameter model containing contracting ventricles based on behavior of sarcomeres, passive atria described with a fixed compliance and resistance, and six compliant vascular compartments. This model was used to understand the behavior of the fetal heart (including its shunts) by changing the arterial pressure, blood volume, heart rate, and afterload (27) and the effect of partial closure of the ductus arteriosus (56). Although the parameters of this model are based on data from fetal lambs, the framework of this model is the basis of multiple mathematical research studies of the fetal cardiovascular circulation. In 1985, Huikeshoven et al. (57) extended their model to 16 compartments and included oxygen transport based on fetal sheep data, but simplified the heart to two chambers and described their function with a cardiac function curve. In addition, they scaled the parameters to human fetal cardiac output, while remaining the same distribution of blood flow to evaluate fetal oxygen consumption (28). One decade later, the first closed-loop lumped parameter model was developed that allowed interpretation of clinical data by verifying parameters of the model on Doppler velocity waveforms of healthy full-term fetuses (29).

In the next decades, the hemodynamics of growth and transition from fetus to neonate were simulated for healthy fetuses (30, 31, 58–60). Pennati and Fumero (59) applied scaling laws to their model (29) in order to evaluate the effect of growth on the pulsatility index in several vessels, on the velocity across the valves, and through the shunts during pregnancy (59). In 2010, Sa Couto et al. (58) studied the hemodynamic effects of the transition from fetus to neonate including the effect of clamping the umbilical cord. This model builds on previously published models of the cardiovascular system of neonates (61–63). With a similar research question and inspired by (29), Yigit et al. simulated the effect of the transition and the effect of cord clamping on the hemodynamics and oxygen saturation of a full-term (30) and preterm fetuses (31). The latter was achieved by scaling the model on GA (64). Similarly, Munneke et al. (60) used CircAdapt (65) to mimic this transition. While the above mentioned models use a time varying elastance model, CircAdapt contains a one fiber model to simulate the contraction of the heart. These models (30, 31, 58–60) show that lumped parameter models can be used to simulate specific events (e.g., transition from fetus to neonate or fetal growth) and evaluate the new distribution of blood flow, pressure, volume, and oxygen saturation. However, when evaluating the model results, it should be taken into account that only spatial average effects can be modeled and that wave propagation and reflections are not taken into account, which could affect the pulsatility index of the flow in the arteries and, in particular, the umbilical arteries.

**Figure 3:**
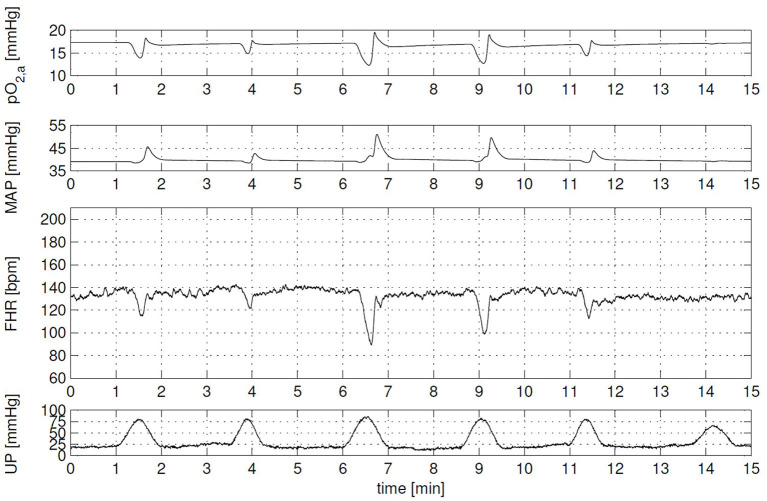
Clinical scenario: cardiotocography (CTG) with variable decelerations and corresponding arterial oxygen (*pO*_2, *a*_) and mean arterial pressure (MAP) signal. Each contraction evokes a different fetal heart rate (FHR) response, due to contraction-to-contraction changes in both uterine pressure level (UP) and duration, and in the compressibility of the umbilical cord. Overall, FHR corresponds well with changes in MAP and *pO*_2, *a*_. This figure is obtained from the Ph.D. thesis of (73) with permission.

During the same time period, the effect of labor on the cardiovascular system including oxygen transport has been an important topic of several research papers. Especially, the effect on the fetal heart rate, because the timing of fetal heart rate decelerations give an estimation of the physiological state of the fetus and it can be measured by the means of cardiotocography. Van der Hout-van der Jagt et al. (52) developed the first model to investigate the underlying mechanisms of the effect of labor on fetal heart rate focusing on early decelerations (52) and late decelerations (66). In addition, van der Hout-van der Jagt et al. investigated fetal oxygenation changes caused by umbilical cord compression (67) and maternal hyperoxygenation affected by labor (68). These models contain the mother and fetal cardiovascular circulation connected through the placenta, fetal-maternal gas exchange (53), and baro- and chemoreceptor reflex (69). Jongen et al. reduced the complexity of these models (52, 66, 67) to limit the number of indirect measured fetal parameters (70) and to study umbilical cord compression during contractions of the uterus (71). In addition, Wang et al. (72) extended the model of (52) to simulate glucose, lactate, and pyruvate to detect fetal acidemia during labor. As far as we know, this model with all these physiological functions (hemodynamics, oxygen transport, baro- and chemoreceptor reflex, and acidemia) describes the most relevant physiological functions integrated in one model so far. An example of a simulation of the effect of uterine contraction (UP) on the fetal heart rate (FHR), mean arterial pressure (MAP), and arterial oxygen pressure (*pO*_2, *a*_) can been seen in Figure 3 (73).

In 2014, Luria et al. (74) and Garcia-Canadilla et al. (75) presented patient-specific fetal cardiovascular models by using Doppler velocity measurements to find hemodynamic differences between healthy and growth restricted fetuses. Luria et al. (74) developed a closed-loop lumped parameter model and fitted the velocities of the model in the umbilical arteries, middle cerebral artery, ductus venosus, tricuspid valve, and mitral valve on corresponding Doppler measurements from healthy and growth restricted fetuses. Furthermore, the model uses scaling laws to allow simulation of fetuses from 21 to 40 weeks (74). Their research output is the comparison of the simulated cardiac output, placental and cerebral blood flow, and percentage of cerebral cardiac output of healthy with growth restricted fetuses. Garcia-Canadilla et al. (75) developed an open-loop model to explore the effect of cerebral vasodilation and placental resistance on the aortic isthmus flow to investigate the relation between aortic isthmus flow and fetal growth restriction. This model is a lumped parameter model that describes in detail the vessels around the aortic isthmus (75) and the umbilical arteries (76) and lumps the rest of the cardiovascular system. The aortic and pulmonary artery inflow are obtained from measured Doppler velocities. The parameters of this model are first tuned to mimic healthy fetuses and subsequently verified on fetuses with fetal growth restriction (75, 76). In addition, this model has been used to evaluate the differences of fetal hemodynamics from diabetic and non-diabetic mothers (77).

##### Placenta

Other studies focus on placental perfusion. The precise cause of placental insufficiency is still unknown, but it is a continuation of an increase in placental resistance due to remodeling of the villous capillary tree leading to obliteration (78, 79). It can be indirectly detected by the abnormal Doppler waveforms in the umbilical artery (78) and/or uterine artery (79). Hence, mathematical models investigate the effect of placental resistance on these indices (80–84). Reuwer et al. (80), Thompson and Stevens (81), Thompson and Trudinger (82), and Guiot et al. (83) focused mainly on the morphology of the feto-placental circulation. The order of branching varies in these studies, but they all investigated the effect of the resistance of the placental capillary bed on the pulsatility index in the umbilical artery. Reuwer et al. (80) studied this effect by the ratio of the resistances of the umbilical arteries and the placental capillary bed and the fetal aortic pulse pressure. Thompson and Stevens (81) and Thompson and Trudinger (82) considered two orders of vessel branching in the placental bed and analyzed the effect of radial changes of the terminal branches. Guiot et al. (83) investigated 15 generations of branching to asses the influence of the ratio of the area of successive order branches of the feto-placental vasculature on pulsatility indices. To analyze the influence of the resistance of different vessels (uterine radials, spiral arteries, and internal cotyledons supplying myometrium and endometrium) in different layers of the placental bed on the uterine waveform, Talbert (84) developed a mathematical model that includes these vessels.

##### Ductus venosus

Pennati et al. (85) focused on the distribution of umbilical venous flow to the ductus venosus. In this study, the function of the ductus venosus was assessed as this shunt provides the heart, *via* the foramen ovale, and subsequently the rest of the system oxygenated blood. With a mathematical model, they analyzed the amount of blood flow that is shunted from the umbilical vein over increasing GA and noticed a decrease in flow.

##### Aorta isthmus

As parameter estimation is challenging, Grigioni et al. (86) introduced a genetic algorithm (87) to fit model variables to physiological values obtained from literature. This closed-loop lumped parameter model contains two ventricles described by time varying elastance models and the purpose of this study was to examine the role of the aorta isthmus. The authors claim to have developed the first model that can simulate the physiological conditions of the aortic isthmus (86).

#### Transmission line models

Transmission line models are mainly used to investigate Doppler indices as wave transmission is important to take into account for simulating wave profiles. These models represent the entire circulation (closed-loop) (88, 89) or a specific vessel, such as a uterine artery (90, 91), placental circulation (92), or a umbilical artery (35, 36).

Mo et al. (90) and Adamson et al. (91) examined the uterine velocity waveform with a transmission line model. This model contains a terminal load to present the distal resistance, and a constant pulsatile flow waveform as input was used (90, 91). Mo et al. (90) examined the dicrotic notch over GA and wave reflection due to reduction of flow. Adamson et al. (91) studied the effect of placental resistance, uterine radius, and mean arterial blood pressure on the uterine waveform.

Also, Todros et al. (92) studied the waveform of the uterine artery and used the placental vascular circulation lumped parameter model of (83) and transformed it to a transmission line model to account for wave reflections. After verifying the model in physiological conditions, pathological conditions concerning restriction in vascular growth, occlusion of vascular bed, and reduction of vascular lumen were examined and their effect on the uterine pulsatiliy index was investigated (92).

Hill et al. (35) and Surat and Adamson (36) designed a model representing the umbilical artery. The inputs of this model are experimentally measured pressure waveforms of fetal sheep. Hill et al. (35) changed the wall properties of the umbilical artery and the location of the terminal bed in order to better understand the hemodynamics of the umbilico-placental circulation. Furthermore, pathological situations where examined by locally occluding placental arterioles with embolization, and following infusion of Angiotensin II to induce vasoconstriction (35). Surat and Adamson (36) added a bifurcation to the model of (35) and verified the healthy situation on the same experimental sheep data. Furthermore, they investigated the influence of attributes of the umbilical artery (altering radius, arterial wall stiffness, and elastic modulus) and placental microcirculation (increased placental resistance) on the umbilical artery flow velocity and Doppler indices (36).

Myers and Capper (88) and Capper et al. (89) both developed a flow-driven closed-loop fetal cardiovascular model to investigate influential factors on Doppler indices in various vessels. Myers and Capper (88) studied the effect of placental resistance on forward and backward traveling waves in relation to pulsatility and resistance index. The simulated Doppler indices based on a healthy placenta were verified with literature values (88). Capper et al. (89) focused only on the pulsatility and resistance index in the umbilical artery and the effect of GA on those indices.

### 1D models

In order to simulate the propagation of pressure and flow waves through the cardiovascular system or compressible fluid, 1D (wave propagation) or higher dimensional models are required. Potentially, series of 0D compartments could simulate wave propagation in a vessel, using a transmission line set-up. However, non-linear convective accelerations are typically not accounted for in transmission line models. As 2D and 3D modeling are computational expensive, 1D modeling is in this case ideal to simulate wave transmissions in vascular networks with acceptable accuracy (48). The identified fetal 1D wave propagation models describe a part of the fetal circulation to answer a specific research question. Similar as lumped parameter models, wave propagation models focus mainly on fetal-placental circulation to understand the underlying mechanism of placental insufficiency and/or to study pulsatility indices. The next paragraphs provides an overview of the wave propagation models and Supplementary Table 2 gives an overview of the assessment based on the technical requirements.

Guettouche et al. (39) and Menigault et al. (42) developed both a mathematical model describing the fetal arterial system from heart to placenta to understand the underlying mechanisms of the fetal placental circulation. Guettouche et al. (39) were the first to apply 1D modeling to analyze fetal-placental circulation and they developed a model consisting of 16 segments. The diameter and length of these segments were defined based on two-dimensional imaging. At the inlets, a velocity profile was imposed described by Fourier coefficients based on Doppler measurements. At the terminals, resistances where applied to represent the rest of the fetal body (39). These resistances were optimized based on experimental measurements by minimizing the difference between the simulated and measured velocities (39, 40). The model of (42) consists of 6 segments from fetal heart to the placenta and one segment (representing the uterine arteries) from maternal side to the placenta. The input at the fetal side is described by a time varying elastance model to mimic the contraction of the heart. At the entrance of the uterine arteries segment, a prescribed pressure is imposed. This model, describing a healthy fetus, is verified with blood velocity profiles, and subsequently used to simulate placental disease by increasing the umbilical resistance (42).

During pregnancy, remodeling of utero-placental vessels is important for optimal exchange of compounds between mother and fetus. Therefore, failure of this transformation could cause maternal hypertension and placental insufficiency (93). Sengupta et al. (34) investigated the conversion from a spiral to a straight utero-placental vessel and simulated the progress of one spiral vessel during pregnancy in normal and abnormal condition by analyzing different geometries of this vessel. The results of abnormal and normal condition were compared with each other and with fetal Doppler recordings of normal and hypertensive pregnant women. Sengupta et al. (34) concluded that placental insufficiency appears in the presence of tapering vessels or local constrictions.

Kleiner-Assaf et al. (33), Hellevik et al. (44), Van den Wijngaard et al. (41), and Sled et al. (32) investigated influential factors on Doppler indices. Kleiner-Assaf et al. (33) analyzed the effect of anatomic and physiological characteristics, and blood properties of the umbilical arteries on different Doppler indices (pulsatility and resistance index and systolic-to-diastolic ratio) with a mathematical model describing an umbilical artery with prescribed pulsatile pressure at the inlet. Furthermore, the effect of the longitudinal and radial measurement location on Doppler indices and mean flow were investigated (33). Although the umbilical vein has a nearly flat profile over time in healthy pregnancy, pulsations in the umbilical vein may occur during early pregnancy and pathological conditions. Hellevik et al. (44) analyzed the pulsatility in the umbilical vein by simulating the hemodynamics in the umbilical vein and ductus venosus bifurcation, by studying the effect of the stiffness of umbilical vein and the ductus venosus-umbilical vein diameter ratio on pulsatility index (44). Van den Wijngaard et al. (41) studied the pulsatility index in several vessels of a fetus growing from 15 to 40 weeks with a model describing the arterial side of the fetus with 13 arterial segments and windkessels at every terminal. Furthermore, the effect of arterial stiffness, blood viscosity and brain resistance on the indices was assessed for a fetus of 40 weeks of gestation (41). Agreeing with the results of (41), Sled et al. (32) pointed out the variation of pulsatility along the umbilical artery. Therefore, Sled et al. (32) investigated wave reflections along the umbilical arteries in normal human pregnancies to potentially explain this variation. During this study, blood velocity waveforms at three different locations along the umbilical arteries were recorded from pregnant women and variation in pulsatile wave profiles along the vessel was analyzed and explained by reflection (32).

In 2011, Mynard (94) described the development of a 1D closed-loop adult cardiovascular system containing 186 vessels. The arterial and the venous side are coupled with lumped parameter elements that represent the microcirculation of organs. Subsequently, he scaled this model to a neonatal cardiovascular system and added 12 vessels to create a fetal cardiovascular system. Both models contain time varying elastance models to simulate the contraction of the heart, a specific coronary circulation lumped parameter element that contains subepicardial and subendocardial layers, and a heart valve model that describes the dynamic motion of the valves. This model (94) was modified to mimic the fetal circulation with dextrotransposition of the great arteries (95) to assess the hemodynamics this pathological condition.

## Discussion

This study explored fetal circulatory models in order to potentially adopt (parts of) existing models to create the digital twin of a fetus of a PLS system and to identify functionalities that still have to be developed. In general, the identified models were able to simulate fetal functionalities realistically, such as presented in Figure 3. Technical requirements (Section Technical requirements) of the digital twin were defined to satisfy predefined functional requirements (Section Functional requirements). Based on these technical requirements, the models included in this study are discussed in the following sections.

### Model assessment

#### Cardiovascular system

The model of (94) presents the only closed-loop fetal cardiovascular model with a complete 1D vessel structure to assess wave propagation and reflections in larger vessels. This model (94) describes a full-term healthy fetus and is adapted by (95) to assess the hemodynamics of fetuses with dextrotransposition of the great arteries. Due to limited hemodynamic data from fetuses, it is not possible to perform model verification for all parameters and variables involved, for instance regarding in the arms and legs. In addition, the vessels in the arms and legs are clinically not relevant to estimate the wellbeing of the fetus in comparison with, for instance, the umbilical vessels or aorta. Therefore, one could argue that those vessels should be simplified by lumping.

#### Closed-loop

Except for (94) and (95), all other closed-loop models [e.g. (27–29, 31, 52, 56–58, 60)] are 0D models and choices for lumping are based on the specific research question. These models give a good general description of the fetal circulation [e.g., (27–29, 56, 57)] and, especially, the global hemodynamic effect of specific events, such as labor [e.g., (52)] or the transition from fetus to neonate [e.g., (31, 58, 60)]. Although several models allow simulation of fetal hemodynamics of different GAs (31, 59, 74), none of these models simulate the transport of oxygen in combination with fetal growth.

#### Pulsatile driving force

To introduce hemodynamic pulsatility, several articles adapted the classic time varying elastance model approach (11) and considered dissipative viscous characteristics of the myocardium (29, 59, 95), interaction of the septum (94), and/or pericardial constraint (94). Considerable advantage of time varying elastance model is its simplicity and, therefore, its straight forward numerical implementation. The one fiber model describes the underlying phenomena of cardiac contraction better. Therefore, it is able to simulate pathophysiology and adaptation on physiological principles. Furthermore, the parametric input is measurable. However, the dependency of the sarcomere shortening velocity makes numerical implementation of this model more challenging since numerical oscillations may occur with small simulation time steps. Van der Hout-van der Jagt et al. (52) as well as the follow-up studies (66–68, 72) describe only the combined left and right ventricular function using a single chamber one fiber model. Only the fetal cardiovascular model including oxygen transport of (60) has a four-chamber one fiber model. Models that impose a prescribed pressure or flow profile obtain this profile either by a simple function [e.g., (80, 82, 92)] or from patient data [e.g., (74, 76)]. When using patient data, the prescribed pressure or flow profile could be used to find parametric differences between healthy and pathological cases by fitting the suspected influenced parameters to the prescribed profile (75–77). In addition, a patient-specific model can be obtained when using measured blood velocity profiles (74).

#### Maternal-fetal gas exchange

Of the models found in literature, only closed-loop lumped parameter models [e.g., (28, 30, 52, 60, 70)] contain simulation of distribution of oxygen. However, none of these models contain fetal growth. The oxygen concentration in an organ is computed over a cardiac cycle. Therefore, non-pulsatile pressures and flows are sufficient and the simulation of wave propagation is not required to compute the saturation of oxygen. Hence, lumped parameter models are the right tool for answering research questions regarding distribution of oxygen. Yet, it is possible to include oxygen distribution in 1D models (96), but it has not been applied in fetal 1D models.

#### Baro- and chemoreceptor reflex

The regulatory system is not only important to maintain homeostasis, but also during fetal distress. For instance, brain sparing response is a mechanism to maintain the oxygen concentration in the brain during fetal hypoxia. Hypoxia triggers mainly the carotid chemoreflex resulting in a decrease of fetal heart rate and an increase of peripheral resistance by vasoconstriction (97). Only (52) and studies using this model (66–68, 72) investigated the effect of acute hypoxia on the fetal cardiovascular system due to uterine contractions. This regulatory model is based on the adult regulatory system of (69) and scaled for the fetus. Although this model simulates decelerations in fetal heart rate realistically, verifying this regulatory model is difficult due to lack of experimental data. Developing a module that describes the fetal regulatory system to maintain homeostasis and reacts during pathological situations, such as hypoxia and hypoglycemia, is a major challenge since the underlying physiology is not (yet) completely understood. Furthermore, clinical research studies often cannot be performed for ethical reasons. In addition, these models simulate full-term fetuses based on scaling of the adult regulatory system, not taking into account immaturity of the fetal regulatory system. Moreover, regulatory models for extremely premature infants still have to be developed and, therefore, none of these models consider fetal growth (Supplementary Table 1).

#### Fetal growth

The fetus approximately doubles in size from 24 to 28 weeks GA (26). Hence, its cardiovascular system grows as well to provide the tissue with the required nutrients. Current mathematical models use scaling laws based on fetal weight or GA to scale the resistances and compliances of their model to the desired GA [e.g., (31, 59, 74, 75)]. However, distribution of cardiac output changes over GA. For instance, the placenta receives about 30% of the cardiac output at 24 weeks and 20% at 40 weeks GA (98, 99). The pulmonary and cerebral flow fraction at 20 weeks GA are estimated at 15 and 8%, respectively, and at 40 weeks GA 25 and 35%, respectively (99). Currently, these changes in flow distribution over GA are often insufficiently included in current scaling laws. Including those physiological changes could provide more realistic models leading to more accurate simulations and predictions.

#### Real time

Simulation time is often not mentioned per model. Computational time depends on the model dimensions, size of geometry, the number of time steps, numerical implementation (e.g., sparse matrices or parallelization), and the computational power of the system. After initialization of the model until dynamic steady state has been reached, real-time can be more easily achieved as the previous condition can be used as initial estimation. In addition, metamodeling could be a solution when 3D modeling is required (100).

### Model integration

**Figure 4:**
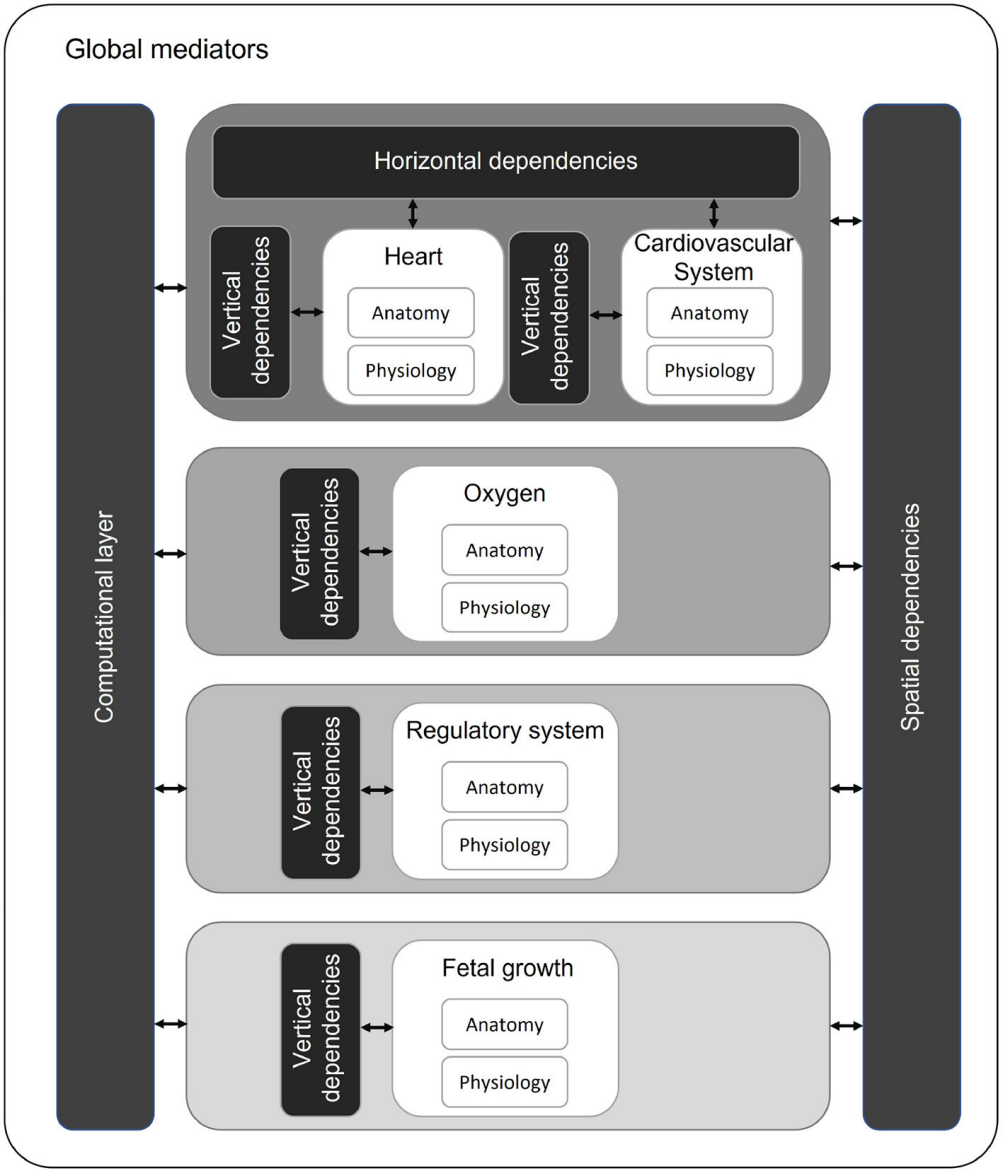
Proposed multiscale and multilevel approach with modules (white) organized on time scales (gray scale). Modules with the same time and length scale can have horizontal dependencies. Within a module, multilevel approach can be required (vertical dependencies). Over different time scales, modules influence each other (spatial dependencies).

This study shows that the existing models are able to capture functionalities of the fetal cardiovascular system including fetal growth and oxygen transport. However, none of the models meet all technical requirements for a digital twin of a fetus in a PLS system. The model of (72) integrated most of the required physiological functions (hemodynamics, oxygen transport, baro-and chemoreceptor reflex, and acidemia) into one system. These functionalities have to be integrated following a multiscale approach since the modules act in different time scales. The contraction of the heart is typically described in about 1,000 time steps per cardiac cycle ($\Delta t=O\left(10^{-4}\right)$ s) to calculate flow and pressure profiles throughout the entire cardiovascular system. The maternal-fetal oxygen exchange is calculated by averaging blood flows and volumes over one cardiac cycle ($\Delta t=O\left(10^{-1}\right)$ s). For the baro- and chemoreceptor reflexes to act (70), it takes up to several seconds ($\Delta t=O\left(10^{0}\right)-O\left(10^{1}\right)$ s) and fetal growth takes place at a time scale of days ($\Delta t=O\left(10^{5}\right)$ s). In similar fashion, the modules are in different length scales as well. Diffusion of oxygen is at cellular scale ($\Delta x=O\left(10^{-5}\right)$ m), while fetal growth is evaluated in centimeters ($\Delta x=O\left(10^{-2}\right)$ m). Integration of these modules at different levels is of importance as the effect of events in the smallest scales have impact on the modules at higher scales. In order to integrate these fetal models based on different time and length scales, we propose a modular approach using the framework suggested by (101) (Figure 4). Every module (white blocks) consists of a separate anatomy and physiology part describing the geometry and physiological parameters of the corresponding module, respectively (Figure 4). These modules are ordered based on their time scale (center, gray scale). Similar as (101), we identify horizontal (same time and length scale) and vertical integration (same time scale and different length scale) of physiological processes. Hence, the modules in the same time scale (e.g., heart and cardiovascular system) depend on each other by means of input and output. For example, the pressure and flow in the vessels of the cardiovascular system depend on the flow over the valves of the heart (horizontal integration). Within a module, multilevel implementation could be required. For instance, the pressure in a heart chamber depends on the sarcomere length (vertical integration). As software structure is based on time loops, there is a spatial dependency between modules. These modules in different time scales will influence each other. For example, insufficient oxygen supply due to placental dysfunction could result in fetal growth restriction (102). The computational layer consists of the mathematical representation of the modules. All these modules, dependencies, and the computational layer are controlled by global mediators. These mediators consists of simulation parameters (e.g., definition of time scales, maximal number of cycles to prevent infinite loops, and convergence tolerance to reach dynamic steady state) and global parameters (e.g., blood density and numerical scheme parameters).

### Model proposal: A digital twin of a fetus in a PLS system

For the purpose of the development of a digital twin of a fetus in a PLS system, the closed-loop 1D fetal cardiovascular model of (94) in combination with a four chamber one fiber model (60) will be the starting point of the development in order to describe the underlying phenomena as realistic as possible. Simplification of model parts will be considered after implementation and analyzing the results. Subsequently, maternal-fetal gas exchange and baro- and chemoreceptor reflexes will be added, using the modular approach described in Section Model integration (Figure 4). Currently, these modules are only found in combination with fetal lumped parameter models. The microcirculation of the model of (94) is lumped. Hence, transport of oxygen (13) can be adopted from lumped parameter models [e.g., (52, 70)]. Comparable, the baro- and chemoreceptor reflexes affect peripheral vascular resistance, cerebral artery resistance, heart rate, venous unstressed volume, and cardiac contraction (70). When corresponding receptors are triggered, the variable or the resistance of the microcirculatory bed can be adjusted accordingly. When these modules are integrated and result in realistic outcomes for a full-term fetus, fetal growth will be incorporated. Fetal growth will be implemented backwards (from 40 to 20 weeks GA) taking into account that the regulatory system for extremely premature fetuses is not completely understood.

### Digital twinning

Integration of the required functionalities leads to a mathematical model that can describe the fetal circulatory system including oxygen transport and fetal growth with respect to the available literature data and retrospective clinical studies for verification. However, to obtain a patient-specific digital twin, the model should respond to measurements of a fetus inside a PLS system (digital twinning). Digital twinning is achieved in two steps, first by identifying the most influential model parameters on simulation outcomes. Preferably, these are the parameters that should be coupled to measurements from the fetus inside a PLS system. In case these required parameter values are immeasurable or unavailable, these parameters can be estimated by comparing model outcome with fetal outcome and/or system behavior. For example, a fetal electrocardiogram gives direct information regarding the heart rate, whereas the placental resistance needs to be estimated from other measurements. Ultimately, the digital twin technology provides additional information that cannot be measured, and the final model can be used for evaluating the effect of possible interventions.

### Limitations

This study summarizes fetal mathematical models satisfying at least one of the technical requirement mentioned in Section Technical requirements. However, this study has some limitations. First, this study does not include models describing amniotic fluid dynamics while fetal growth is likely correlated to amniotic fluid volume, as there is a clear link between birth weight and amniotic fluid volume (103). The amniotic fluid consists grossly of the fetal urine (104). Therefore, amniotic fluid flow including urine production with the renal function parameters (e.g., urea, creatinine, and potassium) should be considered to be included in the technical requirements. Second, metabolism as well as hormone regulation is not incorporated in this study considering the size of these topics. An extensive research should be conducted to map the nutrient utilization and hormone pathways of the fetus and its effect on the other modules. Finally, a specific function of digital twin models is its patient- and situation-specificity, by incorporating measured data. Hence, the models in this study contribute to describing the physiology of the fetus, but the aspect of continuously acting on measured data and predicting the new situation of the fetus is not yet considered. Therefore, an approach that describes the interaction of a digital twin with the real world should be defined as well.

## Conclusion

This study provides an overview of existing 0D and 1D fetal circulatory models. It also proposes a strategy for developing a digital twin of a fetus in a PLS system. Although these models can describe the fetal physiology, this study shows that none of the models meets all the technical requirements for such a digital twin. However, most predefined technical requirements are simulated in different mathematical models and can potentially be incorporated into a new single model. Hence, this study shows the potential to develop a digital twin that meets the functional (Section Functional requirements) and technical (Section Technical requirements) requirements. The challenge for the development of the digital twin is to integrate the desired functionalities of the identified models into a single model, to remain pseudo-real-time with respect to computational time, and to verify the mathematical model with clinical data thoroughly.

## Author contributions

BW wrote the main manuscript text based on a discussion session with all other authors. All authors contributed to the article and approved the submitted version.

## Funding

This project has received funding from the European Union's Horizon 2020 research and innovation programme under grant agreement No. 863087.

## Conflict of interest

MH is shareholder in Juno Perinatal Healthcare BV, Netherlands.

The remaining authors declare that the research was conducted in the absence of any commercial or financial relationships that could be construed as a potential conflict of interest.

## Publisher's note

All claims expressed in this article are solely those of the authors and do not necessarily represent those of their affiliated organizations, or those of the publisher, the editors and the reviewers. Any product that may be evaluated in this article, or claim that may be made by its manufacturer, is not guaranteed or endorsed by the publisher.

## Footnotes

^1^(“Fetus”[MeSH Terms] OR “Parturition”[MeSH Terms] OR “fetus^*^”[Title/Abstract] OR “fetal”[Title/Abstract]) AND (“Hemodynamics”[MeSH Terms] OR “hemodynamic”[Title/Abstract] OR “oxygen^*^”[Title/Abstract]) AND (“models, cardiovascular” [MeSH Terms] OR “Mathematical Computing”[MeSH Terms] OR ((“cardiovascular”[Title/Abstract] OR “oxygenation”[Title/Abstract]) AND “model^*^”[Title/Abstract]) OR ((“mathematic^*^”[Title/Abstract] OR “statistical” [Title/Abstract]) AND “computing^*^”[Title/Abstract])).

## Abbreviations

GA: gestational age
LPM: lumped parameter model
OFM: one fiber model
PP: prescribed profile
PLS: perinatal life support
TLM: transmission line model
TVEM: time varying elastance model.
