# Supplementary material for: A review study of fetal circulatory models to develop a digital twin of a fetus in a perinatal life support system
Source: Front Pediatr. 2022 Sep 21;10:915846. doi: 10.3389/fped.2022.915846 (PMC9532745; doi:10.3389/fped.2022.915846)
Supplement: Supplementary file 1 [file Table_1.pdf]

Supplementary Material

Table S1: Overview of the 0D models. Using background color, the papers are grouped based on Section 2.4. LPM: lumped parameter model, OFM: one fiber model, PP: prescribed pressure or flow profile, TVEM: time varying elastance model.

**Reference**	**Cardiovascular system**	**Closed loop**	**Pulsatile**	**Wave reflections**	**Maternal-fetal gas exchange**	**Fetal growth**	**Baro- and chemoreceptor reflex**
Huikeshoven et al. (1980)	6 compartments	yes	yes	LPM	no	no; 3kg	no
Huikeshoven et al. (1985a)	16 compartments	yes	yes	LPM	yes	no; 3kg	no
Huikeshoven et al. (1985b)	16 compartments	yes	yes	LPM	yes	no; 3kg	no
Pennati et al. (1997)	23 compartments	yes	TVEM	LPM	no	no; 3kg	no
Pennati and Fumero (2000)	23 compartments	yes	TVEM	LPM	no	yes; 20-40w	no
Yigit et al. (2015)	30 compartments	yes	TVEM	LPM	yes	no; 40w and 3.5kg	no
Yigit et al. (2019)	16 compartments	yes	TVEM	LPM	yes	yes; 20-40w	no
Munneke et al. (2021)	12 compartments	yes	OFM	LPM	yes	no; 40w and 3.5kg	no
Sá Couto et al. (2010)	11 compartments	yes	TVEM	LPM	no	no; 3kg	no
Van der Hout-van der Jagt et al. (2012)	9 compartments	yes	OFM	LPM	yes	no; 40w and 3.5kg	yes
Van der Hout-van der Jagt et al. (2013a)	9 compartments	yes	OFM	LPM	yes	no; 40w and 3.5kg	yes
Van der Hout-van der Jagt et al. (2013b)	10 compartments	yes	OFM	LPM	yes	no; 40w and 3.5kg	yes
Bullens et al. (2014)	9 compartments	yes	OFM	LPM	yes	no; 40w and 3.5kg	yes
Wang et al. (2015)	9 compartments	yes	OFM	LPM	yes	no; 40w and 3.5kg	yes
Jongen et al. (2016a)	11 compartments	yes	no	LPM	yes	no; 40w and 3.5kg	yes
Jongen et al. (2016b)	11 compartments	yes	no	LPM	yes	no; 40w and 3.5kg	yes
Luria et al. (2014)	18 compartments	yes	TVEM	LPM	no	yes; 21-40w	no
Garcia-Canadilla et al. (2014)	14 vessels	no	PP	LPM	no	yes; 31–34w	no
Garcia-Canadilla et al. (2015)	19 vessels	no	PP	LPM	no	yes; 28-40w	no

Continued on next page

Table S1: Overview of the 0D models. Using background color, the papers are grouped based on Section 2.4. LPM: lumped parameter model, OFM: one fiber model, PP: prescribed pressure or flow profile, TVEM: time varying elastance model. (Continued)

Kulkarni et al. (2018)	20 vessels	no	PP	LPM	no	yes; 19-35w	no
Reuwer et al. (1986)	1 vessel	no	PP	LPM	no	yes; 16-40w	no
Thompson and Stevens (1989)	Different numbers	no	PP	LPM	no	no	no
Thompson and Trudinger (1990)	Different numbers	no	PP	LPM	no	no	no
Todros et al. (1992)	Different numbers	no	PP	TLM	no	yes	no
Guiot et al. (1992)	Different numbers	no	PP	LPM	no	no	no
Talbert (1995)	7 compartments	no	PP	LPM	no	no	no
Grigioni et al. (2001)	8 compartments	yes	TVEM	LPM	no	no; 3.45kg	no
Mo et al. (1988)	1 vessel	no	PP	TLM	no	yes; 20-25w	no
Adamson et al. (1989)	1 vessel	no	PP	TLM	no	no; 20w	no
Myers and Capper (2000)	9 compartments	yes	PP	TLM	no	no; 28w	no
Capper et al. (2002)	8 compartments	yes	PP	TLM	no	yes; 28-40w	no
Hill et al. (1995)	1 vessel	no	PP	TLM	no	no; 18w	no
Pennati et al. (2003)	4 compartments	no	no	LPM	no	yes; 20-38w	no

Table S2: Overview of the 1D models. Using background color, the papers are grouped based on Section 2.5. LPM: lumped parameter model, OFM: one fiber model, PP: prescribed pressure or flow profile, TVEM: time varying elastance model.

**Reference**	**Cardiovascular system**	**Closed loop**	**Pulsatile**	**Wave reflections**	**Maternal-fetal gas exchange**	**Fetal growth**	**Baro- and chemoreceptor reflex**
Guettouche et al. (1992)	16 segments	no	PP	1D	no	no; 27.5w and 1.25kg	no
Guettouche et al. (1993)	16 segments	no	PP	1D	no	no; 27.5w and 1.25kg	no
Ménigault et al. (1998)	7 vessels	no	TVEM and PP	1D	no	no; 40w	no
Sengupta et al. (1997)	1 vessel	no	PP	1D	no	yes; 3-20w	no
Kleiner-Assaf et al. (1999)	1 vessel	no	PP	1D	no	no	no
Hellevik et al. (2000)	2 vessels	no	PP	1D	no	no; 20-25w	no
Van den Wijngaard et al. (2006)	13 segments	no	PP	1D	no	yes; 15-40w	no
Sled et al. (2019)	1 vessel	no	PP	1D	no	no	no
Mynard (2011)	186 vessels	yes	TVEM	1D	no	no; 40w	no
Zhang et al. (2021)	135 vessels	yes	TVEM	1D	no	no; 40w	no
